# Increased antibiofilm and growth inhibitory effect of Imipenem/Cilastatin nanoliposomes against clinical *Pseudomonas aeruginosa* isolates

**DOI:** 10.1007/s10856-023-06752-0

**Published:** 2023-09-21

**Authors:** Faezeh Milani, Khosro Adibkia, Hamed Hamishehkar, Tooba Gholikhani, Farhad Bani, Morteza Milani

**Affiliations:** 1 Department of Pharmaceutics, Faculty of Pharmacy, Tabriz University of Medical Sciences, Tabriz, Iran; 2 Research Center for Pharmaceutical Nanotechnology and Faculty of Pharmacy, Tabriz University of Medical Sciences, Tabriz, Iran; 3 Drug Applied Research Center, Tabriz University of Medical Sciences, Tabriz, Iran; 4 Department of Medical Nanotechnology, Faculty of Advanced Medical Science, Tabriz University of Medical Science, Tabriz, Iran; 5 Infectious and Tropical Diseases Research Center, and Department of Medical Biotechnology, Faculty of Advanced Medical Sciences, Tabriz University of Medical Science, Tabriz, Iran

## Abstract

Numerous infections are linked to *Pseudomonas aeruginosa*. It is one of the major medical concerns because of virulence and antibiotic resistance. Antibiotic encapsulation in liposomes is a good strategy for controlling infections caused by this microorganism. Evaluation of anti-*Pseudomonas aeruginosa* effect of liposomal form of Imipenem/Cilastatin in vitro condition. By using the disk agar diffusion technique, the isolates’ pattern of antibiotic resistance was identified. The antibiotic was placed into the nanoliposome after it had been made using the thin layer and ethanol injection techniques. SEM and DLS were used to determine the size, shape, and zeta potential of the encapsulated drug form and the empty nanoliposome. Additionally, Imipenem/Cilastatin encapsulation in nanoliposomes was studied using FT-IR spectroscopy. In the microbial assay experiments the MIC, MBC and MBEC of liposomal and free drug forms were determined. The nanoparticles were spherical, with a diameter ranging from 30 to 39 nm, and the EE% in the thin layer and ethanol injection procedures were 97 and 98, respectively. Imipenem/Cilastatin nanoliposomes showed peaks at 3009 cm^−1^ and 1650 cm^−1^, demonstrating the thermodynamic stability for the chemical structure of the drug enclosed and validating the encapsulation of antibiotic in the nanoliposomes. When compared to free drug forms, nanoliposomes had lower MIC and MBC values in the majority of the isolates and had a greater ability to eradicate the biofilm formation. It was shown that the two nanoliposome preparation techniques were more efficient in 80% of the isolates, which had outcomes that were consistent with those of numerous other investigations. Overall, we demonstrated that the antibacterial activity of nanoliposomes was higher than that of the free drug form based on the evaluation of their MIC and MBC. Pharmaceutical nanoliposome techniques provide an excellent future perspective on how to manage microbial infections that are resistant to antibiotics.

**Graphical Abstract:**

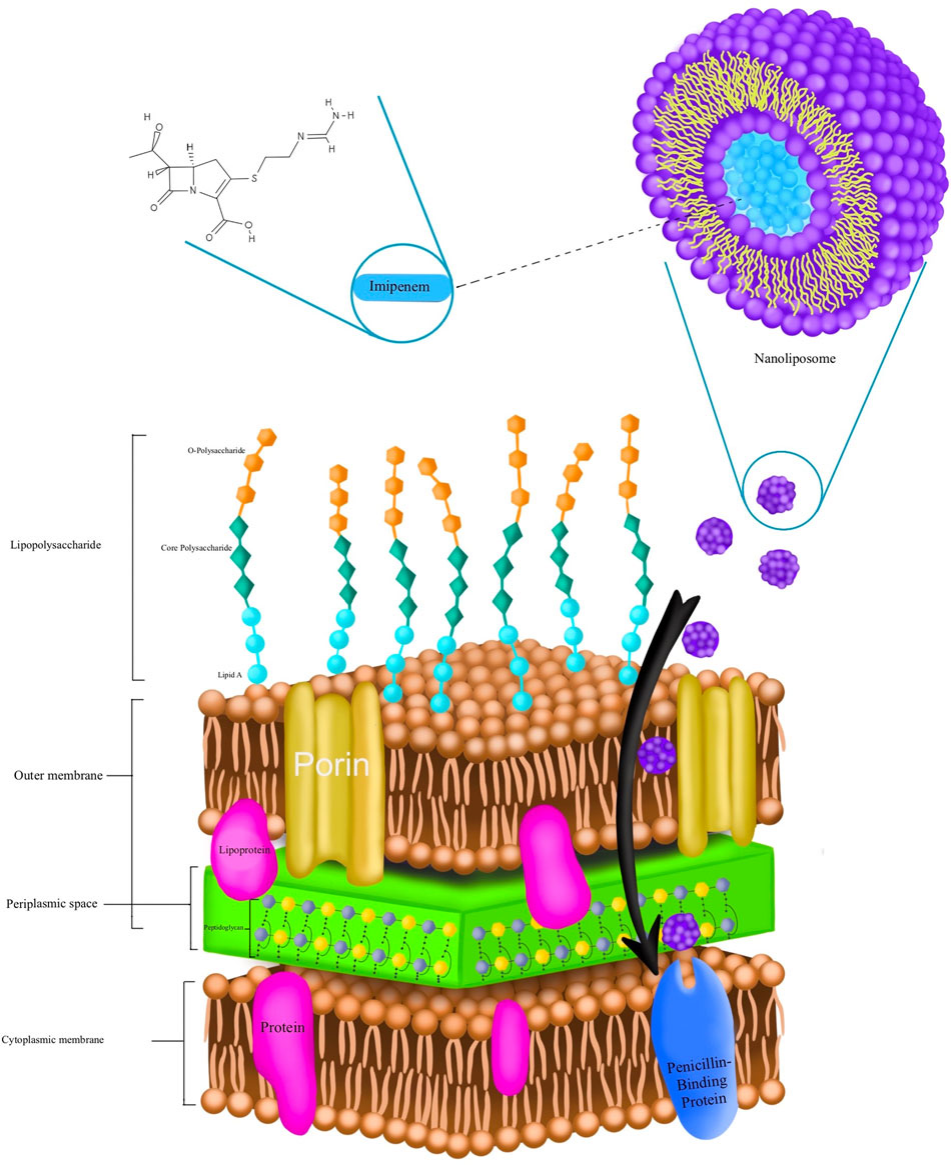

## Introduction

*Pseudomonas aeruginosa* is a gram-negative and opportunistic pathogen related with several human infections [1, 2]. The rise of multi-drug resistant bacteria and decreasing sensitivity make it difficult to control and treat *P. aeruginosa* infections [3]. Through the use of processes such the creation of enzymes, efflux pumps, and alterations to the outer membrane, this bacteria is resistant to antibiotics. Generally speaking, adaptive, inducible, and intrinsic resistance are the three main types of antibiotic resistance [4]. Reduced permeability of the outer membrane, efflux pump expression, and the development of enzymes that render antibiotics inactive are all examples of intrinsic resistance. Resistance is acquired by *P. aeruginosa* through mutation or horizontal gene transfer. The development of biofilm by *P. aeruginosa* contributes to its adaptive resistance by acting as a physical barrier to the bacterial cells’ ability to absorb antibiotics. In fact, biofilms are collections of bacterial cells that produce an extracellular polymeric matrix to shield cells from environmental dangers including the immune system of the host, antibacterial drugs, and nutrition loss [5–7]. It is vital that new antibiotics or other treatment approaches be developed to combat *P. aeruginosa* infections. A combination of penicillin, aminoglycosides, fluoroquinolones, polymixins, and lactamase inhibitors were used for treatment of *P. aeruginosa* infections [5, 8]. Carbapenems such as meropenem and imipenem are among the effective agents used to control *P. aeruginosa* infections, but these antibiotics are also less effective by beta-lactamase producing strains [9–11]. In addition, in recent years the emergence of carbapenems resistant strains related to metallo-beta-lactamaseshas production have been reported. So, it seems that these strains should be considered as microbial agents threatening human health [8, 10]. Inhibition of quorum sensing and bacterial lectins, as well as the use of iron chelation, phage therapy, vaccination approach, nanoparticles, antimicrobial peptides, and electrochemical scaffolds, have all been created as new methods to overcome resistance [12, 13]. Nanoparticles are now widely known for their effectiveness in treating bacterial infections. The nanoparticles may prevent the formation of biofilms, have a high level of penetration in bacterial membranes, and are effective antibiotic transporters. Nanoliposomes are thought to be excellent carriers for antibiotics because they boost medication activity against intracellular and extracellular infections, decrease toxicity, and target drug delivery [14]. The aim of this study was to investigate the anti-*P. aeruginosa* activity of the liposomal form of Imipenem/Cilastatin.

## Material and methods

### Chemicals, bacterial isolates and culture media

Imipenem/Cilastatin was purchased from Afa Chemi Pharmaceutical Company (Tehran, Iran). chloroform (Merck, Germany) Cholesterol was supplied from Merck Chemicals (Darmstadt, Germany), Lecithin was purchased from VAV Lipids Pvt. Ltd (India). Amicon® Ultra Centrifugal Filters, (Merck, Germany) was used. Müller-Hinton agar, Müller-Hinton broth, Tryptic soy broth were purchased from Merck company (Darmstadt, Germany), and Cation-adjusted Mueller Hinton Broth (CHAM) was purchased from Himedia company (India). Nine clinical *P. aeruginosa* isolates were obtained from our previous study [1].

### Synthesis and characterization methods

In this study, nanoliposomes were prepared using two methods including thin layer and ethanol injection. Based on the previous study, the nanoparticle synthesis was done by optimizing this procedure [15].

#### Thin layer method

Briefly, 60 mg of Lecithin was dissolved in 15 ml of ethanol at room temperature, and it was heated under vacuum for 20 min by a rotary evaporator until the solvent evaporates and a thin layer of Lecithin is formed. On the other hand, 10 mg of the drug was dissolved in 2 ml of distilled water and added to the flask containing the Lecithin thin layer in 250 μl volumes. During this step, shaking was continued to load the drug into the nanoliposomes. In the next step, 8 ml of distilled water was gradually added to the flask and heated at 60 °C for 20 min in a rotary evaporator. finally, the falcon containing liposome and drug was placed in ice under sonication (10 cycles of 1 min) to equalize the size of the nanoparticles.

#### Ethanol injection method

In this method, two organic and aqueous phases were prepared separately. To prepare the organic phase, 60 mg of Lecithin was dissolved in 3 ml of ethanol, and to prepare the aqueous phase, 10 mg of the drug was dissolved in 6 ml of distilled water. The aqueous phase was placed in a Falcon homogenizer (200,000 revolutions) at a temperature of 60 °C, and the organic phase was slowly added to the aqueous phase. After mixing the phases, homogenization continued for 20 min. To draw the calibration curve for drug loading capacity calculation, different concentrations of drug (ranging from 150 to 125.3 μg/ml) were prepared and their absorbance was obtained at 320 nm, and the results were plotted as a calibration curve (data not shown). After preparing the nanoliposome, a part of the liposome solution was diluted 1:1 with distilled water and poured into an Amicon filter and centrifuged at 3500 rpm for 10 min. The absorbance of the solution under the filter, which does not contain the loaded drug, was read by a spectrophotometer. Then, the amount of loaded drug was calculated using the calibration curve and the following formula

$${\rm{EE}} \% =\frac{{\rm{Total}}\,{\rm{amount}}\,{\rm{of}}\,{\rm{Drug}}-{\rm{Free}}\,{\rm{amount}}\,{\rm{of}}\,{\rm{Drug}}}{{\rm{Total}}\,{\rm{amount}}\,{\rm{of}}\,{\rm{Drug}}}\times 100$$

$${\rm{LC}} \% =\frac{{\rm{Total}}\,{\rm{amount}}\,{\rm{of}}\,{\rm{Drug}}-{\rm{Free}}\,{\rm{amount}}\,{\rm{of}}\,{\rm{Drug}}}{{\rm{Nanoparticles}}\,{\rm{Weight}}}\times 100$$

Particle size and size distribution were determined by Dynamic Light Scattering (DLS). Also, the shape and surface morphology of the nanoparticles were investigated by scanning electron microscope (SEM). To verify whether Imipenem/Cilastatin was successfully incorporated into the nanoliposomes, FT-IR spectroscopy was accomplished.

### Microbial assay

#### Bacterial isolates and antimicrobial susceptibility testing

A number of clinical isolates of *P. aeruginosa* were acquired from the hospital laboratory, and the disk agar diffusion technique was used to examine the pattern of susceptibility and resistance to popular treatment antibiotics. Nine resistant isolates from among these isolates were used in the investigation. Trypticase soy broth with 15% glycerol and a temperature of −70 °C was utilized to preserve these isolates.

#### Minimum inhibitory concentration (MIC) and minimum bactericidal concentration (MBC) of the free and liposome-encapsulated Imipenem/Cilastatin

In 96-well plates with 150 µl sterile Tryptic Soy Broth medium, serial dilutions of the antibiotic’s imipenem/cilastatin from 250 to 1.9 µg/ml were created for this purpose. The amount of 10 µl of the bacterial suspension equal to 0.5 McFarland was inoculated into each well independently. The plates underwent a meticulous examination to look for signs of bacterial growth suppression following an overnight incubation at 37 °C. Accordingly, each well contained a specific concentration of the drug. The concentration of the drug that could inhibit the growth of bacteria was considered as the MIC. The contents of each well were sub-cultured individually on the Muller Hinton agar medium to determine MBC. The colonies developed on the agar surface were inspected after the plates were incubated at 37 °C overnight. Indeed, the well in which no growth of bacteria was observed in the sub-culture was considered as the MBC. Two prepared forms of Imipenem/Cilastatin nanoliposome (produced by thin layer and ethanol injection technique), were investigated separately against to the tested bacterial isolates, and the outcomes were reported. We employed serial dilutions of nanoliposomes without bacterium inoculation to compare the inhibiting growth of bacteria since the serial dilutions of nanoliposomes exhibited turbidity, preventing macroscopic inspection of the MIC of nanoliposomes.

#### Investigation of biofilm formation in tested isolates

Based on earlier research, 96-well plates were filled with 180 microliters of CAMHB culture medium containing 0.5% glucose and 20 μl of 0.5 McFarland bacterial suspension was added to each well. The contents of the wells were emptied and three times cleaned with 300 μl of sterile phosphate buffer following a 24-h incubation at 37 °C. The wells were filled with 150 μl of methanol and fixed for 20 min at lab temperature. The wells’ contents were then dumped and left to air dry for 30 min at room temperature. One hundred and fifty microliters of crystal violet were added for 15 min at room temperature to stain the material. The dishes were then gently rinsed under running water and dried. After 30 min at room temperature and 150 μl of 33% acetic acid were added to the wells, an ELISA reader was used to read the absorbance at 570 nm. As a negative control, wells with culture media but no bacterial suspension were employed, and acetic acid alone was used as a blank. No biofilm formation (OD ≤ ODc), weak formation (OD ≤ 2ODc), moderate formation (2ODc < OD ≤ 4OD), or strong formation (4ODc < OD) were seen throughout the course of the experiments, which were all run in duplicate.

#### Minimal biofilm eradication concentration (MBEC) of the free and liposome-encapsulated Imipenem/Cilastatin

Except that biofilm development was examined when various drug forms were present, all processes were analogous to the biofilm formation experiment. So that repeated medication dilutions ranging from 250 to 1.9 µg/ml were made after adding CAMHB medium containing 0.5% glucose to the wells. Then, each well received a bacterial suspension, and tests were conducted one at a time. For each isolate, these tests were conducted twice, individually, and with various medication dosages.

## Results

### Characterization of Imipenem/Cilastatin-loaded liposomes

**Fig. 1:**
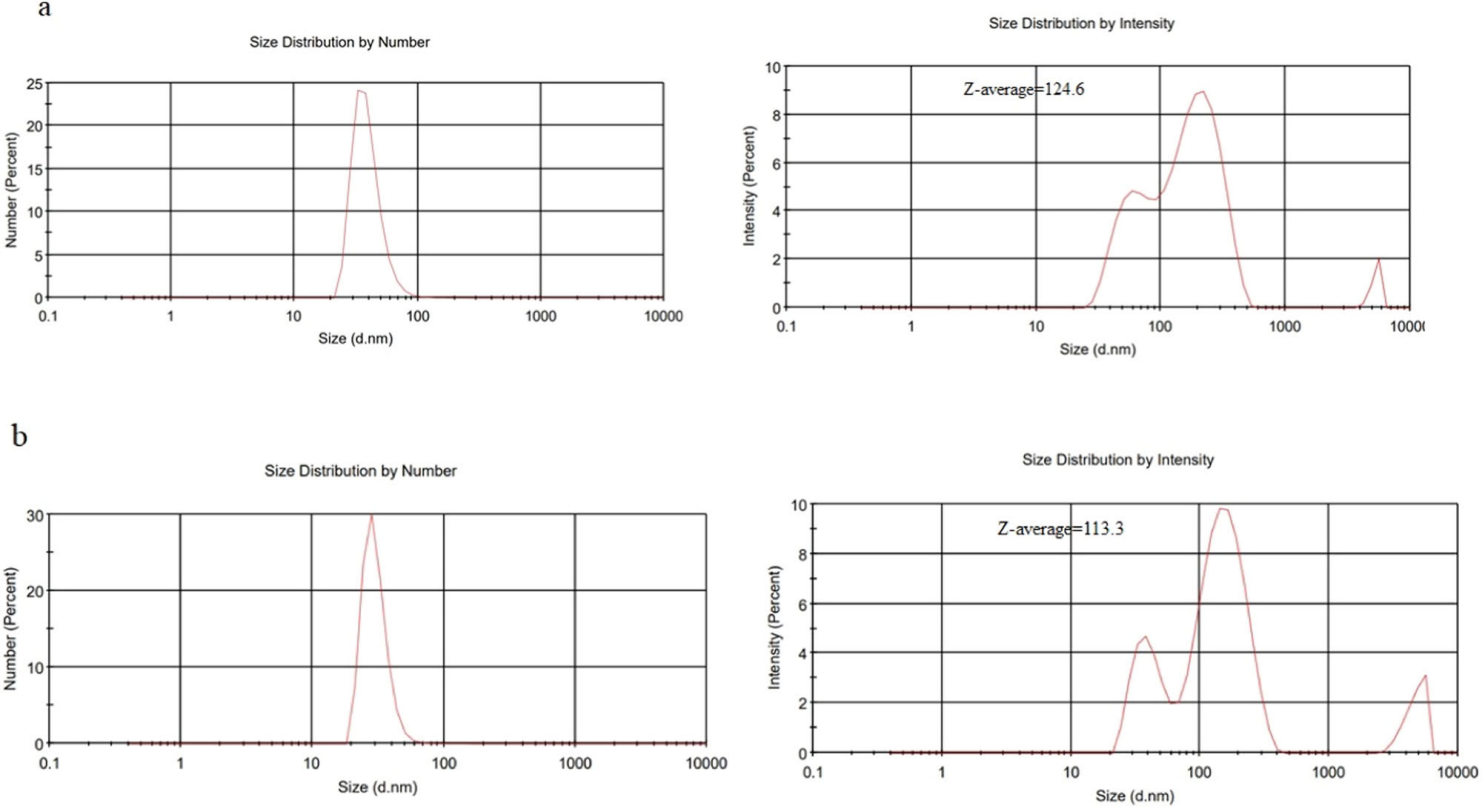
The intensity and number weighted particle size distribution of Imipenem/Cilastatin-loaded liposomes synthesized by **a** thin layer and **b** ethanol injection methods analyzed by DLS

**Fig. 2:**
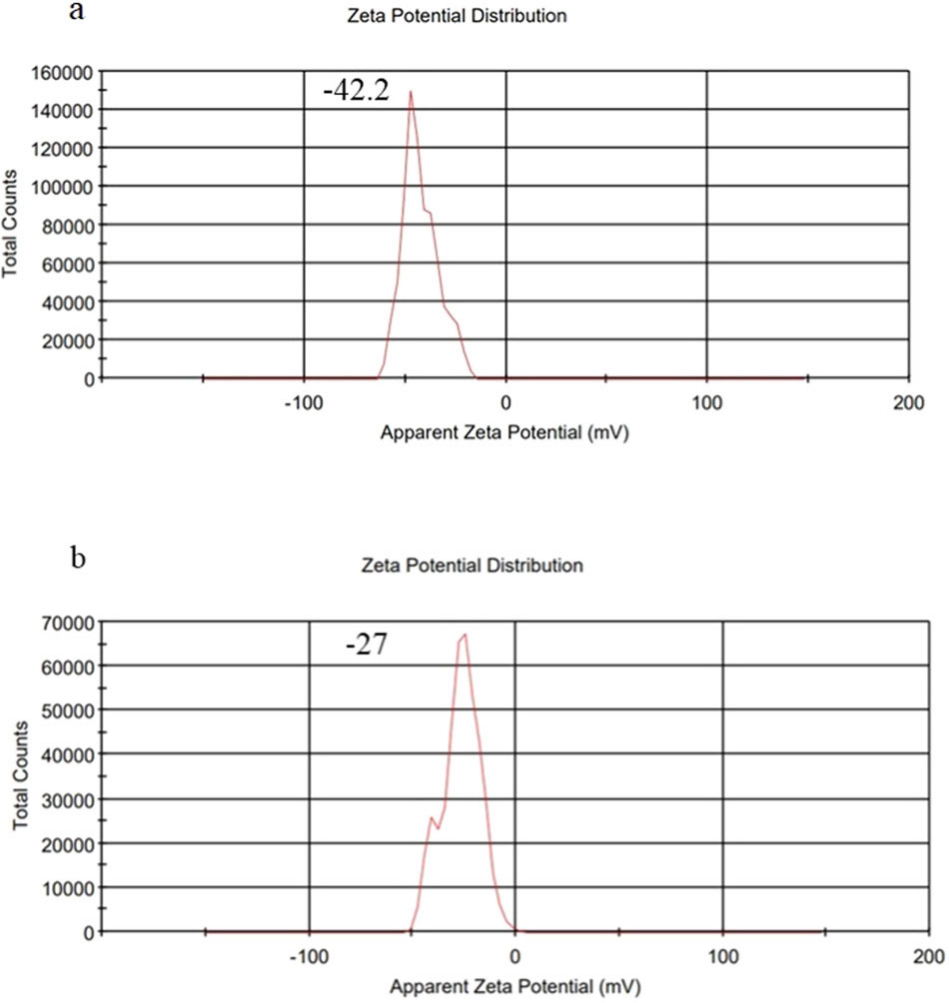
The Zeta potential of Imipenem/Cilastatin-loaded liposomes synthesized **a** thin layer and **b** ethanol injection methods

The findings of the thin layer and ethanol injection techniques for encapsulation effectiveness, loading capacity, particle size, Polydispersity Index (PDI), and Zeta potential distribution were displayed in Table 1. By using DLS, the size of the two techniques used to synthesize Imipenem/Cilastatin-loaded liposomes was examined (Fig. 1). Mean intensity weighted particle diameter and PDI were in the range of 124.6 nm and 0.490, respectively, in the Thin layer technique. Additionally, with the Ethanol Injection Method, these values were 113.3 nm and 0.409, respectively. The number weighted particle size distributions indicated acceptable size distributions for both methods. Additionally, zeta potential distribution was studied by DLS, and the outcomes showed that the mean zeta potential of particles created using the thin layer and ethanol injection procedures, respectively, was −42.2 and −27 mV (Fig. 2).

**Table 1:** Characteristics of nanoliposomes prepared by two methods

Method used	Entrapped Efficiency %	Loading capacity %	Intensity weighted particle size, (nm)	PDI	zeta potential distribution (mV)
Thin layer	96.9%	16.05%	124.6	0.490	−42.2
Ethanol injection	97.8%	16.05%	113.3	0.409	−27

**Fig. 3:**
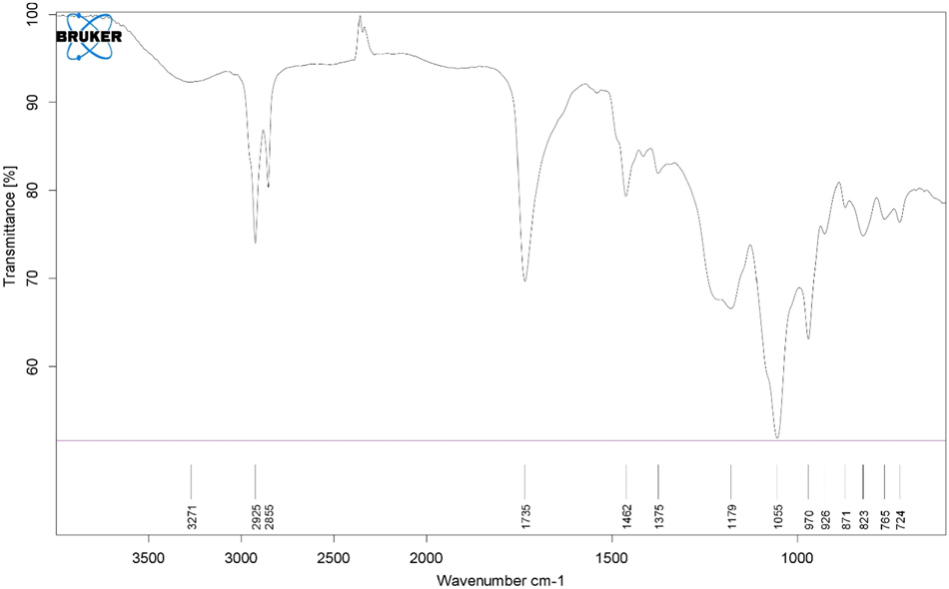
The FT-IR spectrum of nanoliposomes

**Fig. 4:**
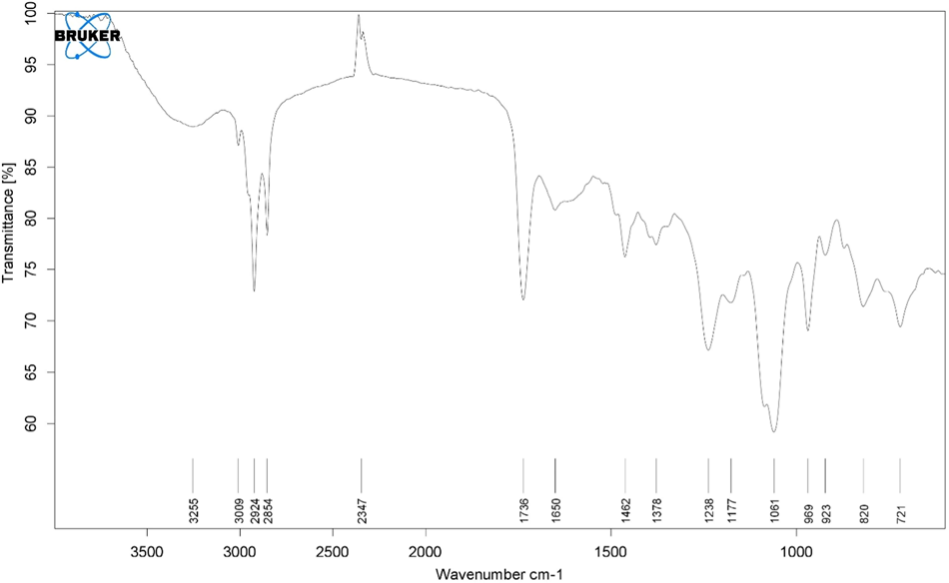
The FT-IR spectrum of the Imipenem/Cilastatin-loaded liposomes

**Fig. 5:**
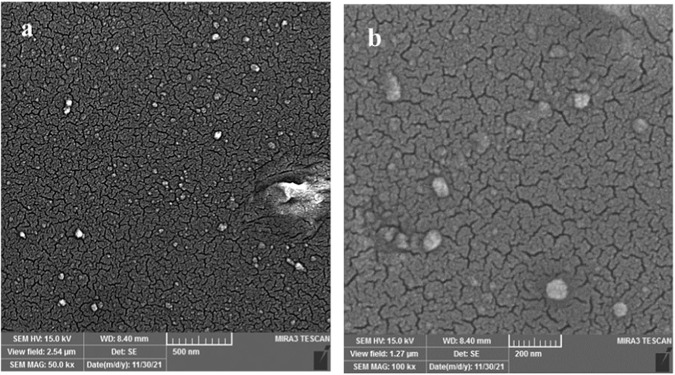
The SEM imaging of Imipenem/Cilastatin-loaded liposomes synthesis by two methods, thin layer (**a**) and ethanol injection methods (**b**)

Imipenem/Cilastatin-loaded nanoliposomes and their FT-IR spectra are depicted in Figs. 3 and 4, respectively. The phospholipids showed their characteristic CH_2_ symmetric and asymmetric stretching vibration peaks at 2925 and 2855 cm^−1^, as well as symmetrical C=O stretching vibration peak at 1735 cm^−1^. When Imipenem/Cilastatin was included in the nanoliposomes, the peak locations and shapes at 2924 cm^−1^ and 2854 cm^−1^ (CH_2_ vibration absorption) and 1737 cm^−1^ (symmetrical C=O stretching vibration absorption) were identical to those of the phospholipids. In the meantime, Imipenem/Cilastatin nanoliposomes displayed peaks at 3009 cm^−1^ (N–H stretching) and 1650 cm^−1^ (amide band), which are the characteristic peaks of Imipenem/Cilastatin. This supported Imipenem/Cilastatin encapsulation in the nanoliposomes and illustrated the chemical structure’s thermodynamic stability. The SEM imaging confirmed that the Synthesized nanoparticles have spherical shape (Fig. 5).

### Minimum inhibition concentration of different drug forms

**Fig. 6:**
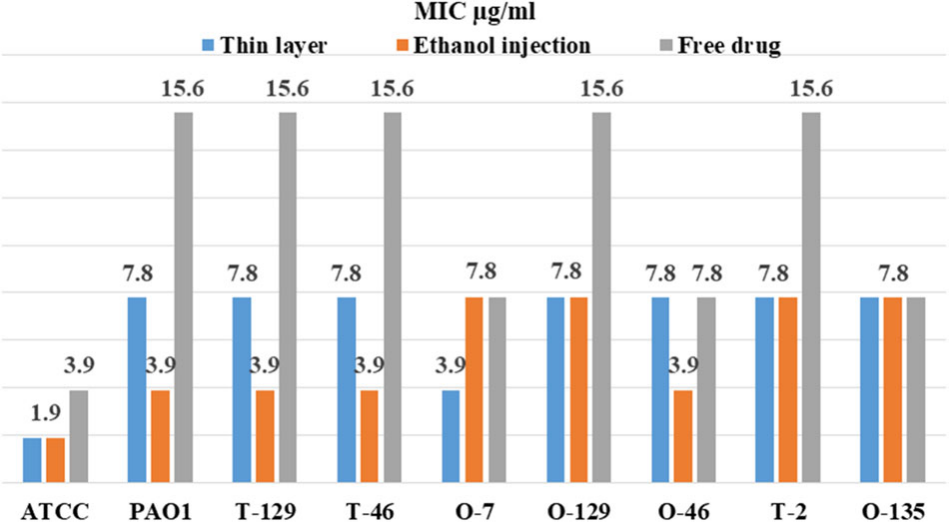
MIC results of Imipenem/Cilastatin-loaded liposomes and free drug form against *P. aeruginosa* isolates

The results of this study showed that the MIC of Imipenem/Cilastatin-loaded liposomes were lower than the free form of antibiotics in ethanol injection and thin layer methods (Fig. 6).

### Minimum biofilm eradication concentration of different drug forms

Our results showed that the liposomal form of Imipenem/Cilastatin has a high capacity to eradicate biofilm formation compared to free drug forms. In general, the antibiofilm activity of the liposomal drug form prepared by the ethanol injection was slightly better than in the thin layer method (Table 2).

**Table 2:** The MBEC results of different drug forms against of *P. aeruginosa* isolates

Bacterial isolates	Drug forms	Drug concentration (µg/ml)							
		250	125	62.5	31.25	15.62	7.81	3.9	1.95
ATCC	Free	0	0	0	0	0	0	w	w
	EINJ	0	0	0	0	0	0	0	w
	TL	0	0	0	0	0	0	0	w
PAO1	Free	0	0	0	0	m	s	s	s
	EINJ	0	0	0	0	0	w	s	s
	TL	0	0	0	0	w	s	s	s
T-129	Free	0	0	0	0	0	w	s	s
	EINJ	0	0	0	0	0	w	w	s
	TL	0	0	0	0	0	0	w	s
T-46	Free	0	0	0	0	0	w	s	s
	EINJ	0	0	0	0	0	0	w	s
	TL	0	0	0	0	0	s	s	s
O-7	Free	0	0	0	0	0	w	w	w
	EINJ	0	0	0	0	0	0	s	s
	TL	0	0	0	0	0	s	s	s
O-129	Free	0	0	0	0	w	w	w	m
	EINJ	0	0	0	0	0	s	s	s
	TL	0	0	0	0	0	s	s	s
O-46	Free	0	0	0	0	0	w	w	w
	EINJ	0	0	0	0	0	w	m	s
	TL	0	0	0	0	0	w	s	s
T-2	Free	0	0	0	0	0	s	s	s
	EINJ	0	0	0	0	0	s	s	s
	TL	0	0	0	0	0	s	s	s
O-135	Free	0	0	0	0	0	s	s	s
	EINJ	0	0	0	0	0	s	s	s
	TL	0	0	0	0	0	s	s	s

*0* no biofilm formation, *w* weakly, *m* moderate, *s* strong

## Discussion

Antibiotic treatment is a branch of medicine that uses antibiotic prescription to prevent and cure infections. To date, many antibiotics have been used for the prevention and treatment of microbial infections. Despite progress in the production and application of antibiotics in human and veterinary medicine, irrational medication prescriptions have led to antibiotic resistance of bacteria. It has been suggested that *P. aeruginosa* is linked to a wide variety of human infections and illnesses due to its innate resistance to the majority of antibiotics. The *P. aeruginosa* infections were successfully managed by broad-spectrum antibiotics imipenem. However, emerging data have indicated carbapenem resistance in infections associated with this bacterium [16–18]. Commensurate with these descriptions, development, and application of novel therapeutic approaches are mandatory. Emerging data have indicated that nanoliposomes are suitable platforms for the prevention and treatment of fatal microbial diseases [19, 20]. For instance, the encapsulation of antibiotics inside the nanoliposomes not only can preserve the loaded substance from bacterial enzymes but also increase the delivery capacity via escaping the physical barriers of the bacterial cell wall. These features make nanoliposomes interesting and suitable therapeutic platforms in bacterial infections. The production of biofilm is helpful in the attachment of several bacteria species to host eukaryotic cells, colonization, and survival in which the inhibition of biofilm synthesis can lead to the reduction of bacterial growth and dissemination [21, 22]. Here, we aimed to examine the bactericidal properties of the Imipenem/Cilastatin-encapsulated nanoliposomes on Imipenem-resistant *P. aeruginosa*. Biofilm eradication, bacterial colonization, and dissemination were monitored in treated isolates. Our data indicated that the designed nanoliposomes exhibited typical physicochemical properties (dimensions, shape, loading capacity, and entrapped efficiency) that were consistent with the previously published data [2, 23]. It was discovered that gentamicin and kanamycin had the greatest and lowest rates of resistance in the examined bacterial isolates, respectively, and that cholestin and piperacillin had the lowest rates of resistance. Additionally, there was 56%, 45%, and 56%, resistance to ceftazidime, imipenem, and ciprofloxacin, respectively. Similar to our study, it was reported that Mushtaq et al. had the lowest sensitivity to gentamicin and imipenem [24]. According to data obtained from this study, the MIC values of Imipenem/Cilastatin-encapsulated nanoliposomes by both techniques were comparable in 5 bacterial isolates. To be specific, the MIC of nanoliposomes synthesized using the ethanol injection method was the lowest in 5 bacterial isolates. It is believed that the genetic diversity of bacterial isolates from clinical samples and the various mechanisms of antibiotic resistance used by bacteria can result in different MIC values after exposure to synthesized nanoliposomes. The fabricated nanoliposomes using both techniques yielded higher antibacterial activity compared to the free antibiotic form. Data confirmed that nanoliposomes exerted bactericidal properties almost in 80% of the sample isolates. Interestingly, the nanoliposomes prepared by the thin layer method compared to the free drug form, reduced the MIC to half the amount in 70% of isolates. While nanoliposomes prepared by the ethanol injection method reduced the MIC to half the amount in 50%, and one-fifth in 40% isolates compared to the free drug form. In an experiment, the antibacterial activity of Imipenem/Cilastatin-encapsulated polymeric nanoparticles was examined against carbapenem-resistant bacteria. It was suggested that the dynamic growth of *P. aeruginosa* and *Klebsiella pneumoniae* strains was significantly inhibited by the polymeric form of the antibiotics compared to the free drug forms [25]. Our earlier work showed that the polymeric form of piperacillin/tazobactam yields prominent antibacterial properties. In other studies, the antibacterial activity of Imipenem in nanoparticle forms was examined against *Staphylococcus epidermidis*. Data revealed the susceptibility rates of MRSA to antibiotics with simultaneous antibiofilm activity [16]. Along with our data, Yuling et al. showed that micelle-loaded triclosan possesses strong biofilm eradication activity [26]. Fonseca et al. proved the inhibitory effects of encapsulated piperacillin/tazobactam on adhesion factors, motility, and biofilm eradication of *P. aeruginosa* strains [23]. In most experiments, the formulations of antibiotics with nanoparticles have boosted antibacterial activity. In the present study, the application of nanoliposomes lowered the MIC and MBC of the antibiotics and blunted the biofilm activity of bacterial isolates.

There appear to be variations between our study and other investigations. Examples include the kind of antibiotic, the types of bacteria, and the kind of nanoparticle. While other research utilized antibiotics including Cefprazone/Sulbactam, Meropenem/Tazobactam, Piperacillin/Tazobactam, Amoxicillin/Clavulanic acid, Ampicillin/Tazobactam, Ticarcillin/Clavulanic acid, and Evibactam/Ceftazidime, we looked at Imipenem/Cilastatin. In some studies, antimicrobial tests have used on other species, and or standard strains, while we used clinical isolates. Of note, *P. aeruginosa* isolates are much more resistant compared to the other strains such as *S. epidermidis*, and naturally higher concentrations of drugs are needed to inhibit them. As a result, it was impossible to compare the findings of this investigation with exactly comparable trials. Antibiotics within the nanoliposomes can exert higher antimicrobial properties, and this approach may be used for the formulation of different antimicrobial drugs and managing microbial illnesses.

## Conclusion

Designed Imipenem/Cilastatin-encapsulated nanoliposomes affected 80% of the *P. aeruginosa* isolates. Compared to the free antibiotic form, treatment of *P. aeruginosa* isolates with encapsulated forma led to the inhibition of biofilm synthesis, and lessening MIC values. It is suggested that antibiotic-loaded nanoliposomes are valid drug delivery routes for several bacterial infections.
